# Development and validation of a machine learning model for predicting coronary artery disease risk: a retrospective study

**DOI:** 10.3389/fcvm.2026.1874227

**Published:** 2026-09-22

**Authors:** Yingying Zhou, Yuxin Chen, Shaoren Rao, Xin Zhang, Junde Fang, Fuqing Guan, Tianfa Li, Jin Peng, Yuanyuan Zhang

**Affiliations:** 1 Department of Cardiology, The First Affiliated Hospital of Hainan Medical University, Haikou, Hainan, China; 2 Department of Cardiology, The 928th Hospital of the Joint Logistics Support Force of the People's Liberation Army of China, Haikou, Hainan, China; 3 Department of Cardiology, Danzhou Western Central Hospital, Danzhou, Hainan, China; 4 Department of Cardiology, The First Affiliated Hospital of Hainan Medical University, Haikou, Hainan, China; 5 Key Laboratory of Tropical Cardiovascular Diseases Research of Hainan Province, Medical Research Center of the First Affiliated Hospital, Hainan Women and Children Medical Center, Key Laboratory of Emergency and Trauma of Ministry of Education, Hainan Medical University, Haikou, China; 6 Department of Cardiology, The First Affiliated Hospital, Hainan Medical University, Haikou, China

**Keywords:** carotid ultrasound, coronary artery disease, Gensini score, machine learning, prediction model

## Abstract

**Background:**

The mortality rate from coronary atherosclerotic heart disease is rising year by year, affecting an increasingly younger population, and is projected to continue rising in the future. Although medical technology is advancing, coronary angiography cannot be widely adopted for screening due to factors such as economic constraints, disparities in regional healthcare standards, and patient compliance. This study aimed to develop and validate a machine learning-based predictive model for assessing the severity of coronary artery lesions and to explore related risk factors.

**Methods:**

This retrospective study included 400 patients who underwent their first complete coronary angiography at the First Affiliated Hospital of Hainan Medical University between 2023 and 2025. The patient dataset from July 2023 to July 2025 (*n* = 300) was randomly divided into an internal training set and a test set in a 7:3 ratio. The temporal validation set consisted of patients (*n* = 100) from the same center between August and December 2025. Key feature variables were used to construct predictive models using eight machine learning algorithms, including Extreme Gradient Boosting (XGBoost), Logistic Regression (LR), Random Forest (RF), Support Vector Machines (SVM), K-Nearest Neighbors (KNN), Artificial Neural Networks (ANN), Naive Bayes (NB), and Gradient Boosting Machines (GBM). Model performance was analyzed using receiver operating characteristic (ROC) curves, 10-fold cross-validation, and decision curve analysis (DCA). The optimal model was validated on the temporal dataset, and its interpretability was assessed using SHapley additive explanation (SHAP). The model was further successfully implemented as a web application using the Shiny package (version 1.13.0) in R 4.5.2.

**Results:**

Ten predictors were retained. The XGBoost model exhibited the best discrimination, with AUCs of 0.889 (95% CI: 0.819–0.945) in the internal test set and 0.803 (95% CI: 0.718–0.888) in temporal validation. The model also showed good calibration and low Brier scores, with stable performance in ten-fold cross-validation. DeLong's test confirmed its superiority over competing models. DCA and SHAP analysis further supported its clinical utility and interpretability.

**Conclusion:**

This study developed a machine learning model for predicting the severity of coronary lesions, demonstrating satisfactory performance in a single-center setting.

## 1 Introduction

Cardiovascular Disease (CVD) is a major and growing global public health burden, among which coronary artery disease (CAD), as one of the most common and fatal diseases, has a continuously rising incidence and disease burden (1, 2). In recent years, clinical research on coronary heart disease has made significant progress in diagnostic techniques, treatment strategies, risk stratification, and management of special populations. Although coronary angiography is the “gold standard” for diagnosing CAD, its invasive nature, high cost, and certain operational risks make it difficult to be widely used for population screening. This results in a lack of efficient and safe assessment methods for identifying and stratifying high-risk populations in the early stages of the disease, thereby preventing the effective prevention of adverse cardiovascular events (3).

Carotid and coronary atherosclerosis share high pathophysiological homology. Increased carotid plaque area (CTPA), carotid intima-media thickness, plaque number, plaque burden, and stenosis severity are all associated with higher prevalence of CAD, more diseased vessels, and higher Gensini or SYNTAX scores (4–6). Currently, the assessment of carotid artery lesions relies on various non-invasive imaging techniques, among which ultrasonography has become the preferred and most commonly used screening and assessment tool in clinical practice due to its simplicity, lack of radiation, good reproducibility, and relatively low cost (7, 8).

Machine Learning (ML), an emerging branch of Artificial Intelligence (AI), excels at integrating diverse population characteristics to fit better predictive models and has been widely applied to medical data analysis in recent years (9, 10). Machine learning algorithms have incomparable advantages over traditional statistical methods in processing high-dimensional data, identifying complex non-linear relationships, and discovering hidden patterns, which demonstrates their great potential in CAD risk prediction (11, 12). Another advantage of machine learning models is their ability to automatically select the most predictive features from a large number of candidate variables to build more concise and efficient predictive models (12). Compared with traditional regression models, machine learning models can integrate more types of predictive variables, including clinical data, imaging features, biomarkers, genomic data, etc., thereby constructing more comprehensive risk prediction models (11, 13).

In this three-year retrospective cohort study, we aimed to develop and validate a machine learning model for predicting coronary artery lesions by integrating carotid ultrasound data. We evaluated eight machine learning algorithms, including eXtreme Gradient Boosting (XGBoost), Logistic Regression (LR), Random Forest (RF), Support Vector Machine (SVM), K-Nearest Neighbors (KNN), Artificial Neural Network (ANN), Naïve Bayes (NB), and Gradient Boosting Machine (GBM). In this study, model performance was validated through ten-fold cross-validation, and the potential clinical utility of the model at different threshold probabilities was evaluated using Decision Curve Analysis (DCA). The high efficiency of the model was further confirmed through temporal validation.

## 2 Methods

### 2.1 Populations

The study data were obtained from the First Affiliated Hospital of Hainan Medical University, collected through a historical cohort between 2023 and 2025. The study enrolled 400 inpatients: 300 in the internal validation cohort (157 high-risk) and 100 in an independent temporal validation set of consecutive subsequent patients (52 high-risk).

Inclusion criteria: 1) age >18 years; 2) patients who completed both carotid color Doppler ultrasound and coronary angiography during hospitalization; 3) for patients with multiple admissions, only the first eligible record was included in the analysis. Exclusion criteria: 1) patients aged <18 years; 2) patients who did not complete both carotid ultrasound and coronary angiography during hospitalization; 3) patients with advanced malignancy or a history of major surgery within one year. Ultimately, missing data were excluded through manual case-by-case verification; no form of imputation was performed. Figure 1 illustrates the design flowchart of this study.

**Figure 1:**
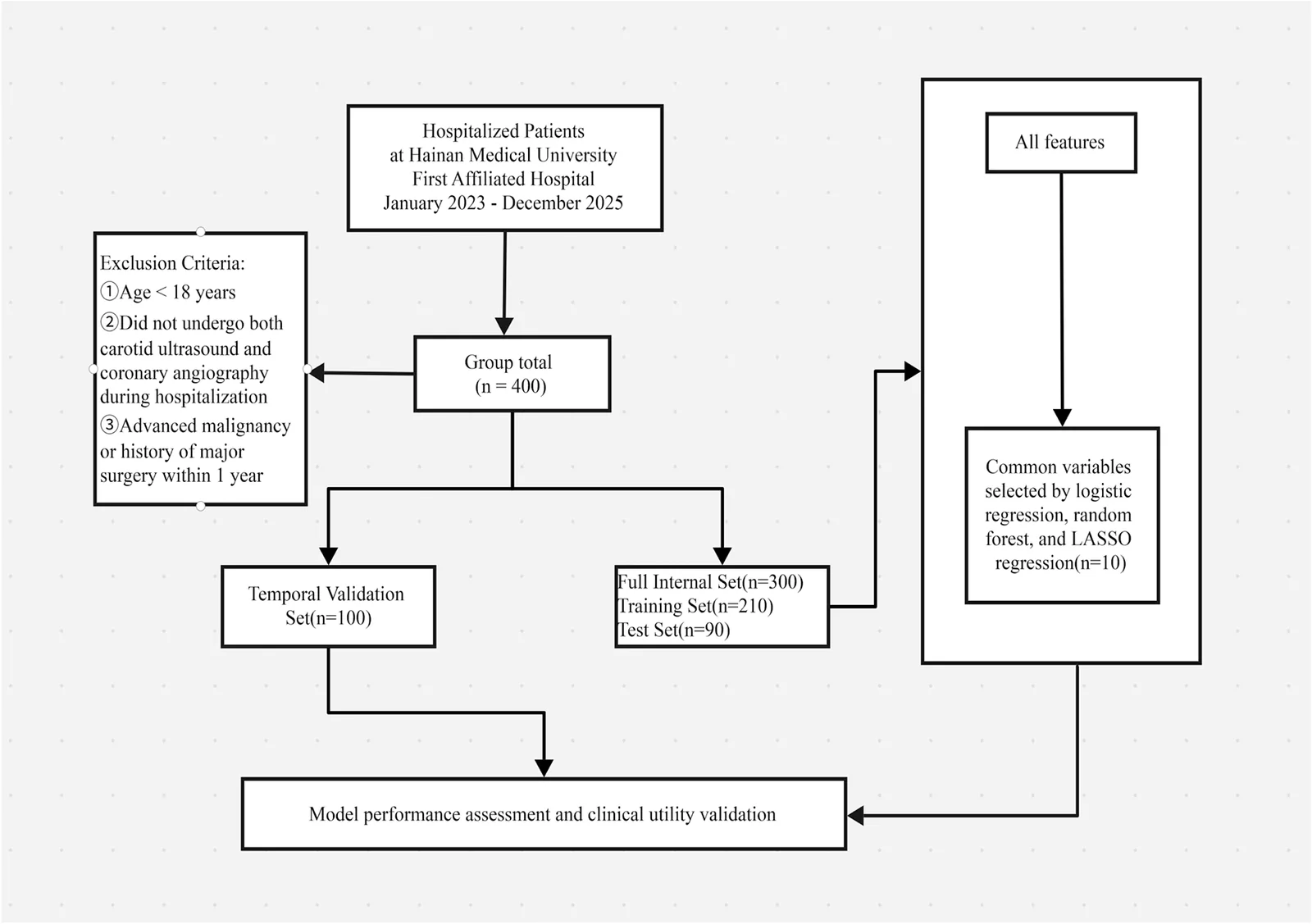
Technical roadmap flowchart.

Relevant patient data were collected from the hospital medical record system in 2025, and the study was approved by the ethics review board (Approval No.: 2025-KYL-241). The design, implementation, and reporting of this study adhered to the Transparent Reporting of a Multivariable Prediction Model for Individual Prognosis or Diagnosis (TRIPOD) recommendations, with specific reference to the TRIPOD+AI extension for machine learning-based prediction models (14, 15).

### 2.2 Clinical implications of Gensini score

Logistic regression analysis showed that the Gensini score was independently associated with stent implantation (defined as a stenosis rate >75% in a single vessel) (OR = 1.07, 95% CI: 1.05–1.08, *P* < 0.001), indicating a dose-response relationship between the severity of angiographic findings and this clinical endpoint.

### 2.3 Variables and data definition

Univariate logistic regression analysis was performed on 56 enrolled variables (Including traditional risk factors such as age, sex, hypertension, diabetes, and hyperlipidemia), with *P* > 0.1 as the elimination criterion, resulting in 22 retained variables. After cross-validation using multiple algorithms, some traditional risk factors did not meet the inclusion threshold for feature importance, possibly due to multicollinearity or medication history affecting traditional risk factors such as blood lipids. We prespecified a threshold of Spearman correlation coefficient |*r*| > 0.8 as the criterion for excluding collinear variables; however, in the actual analysis, no pair of variables exhibited a correlation exceeding this threshold, and thus no variables were removed on this basis, see Figure 2A.

**Figure 2:**
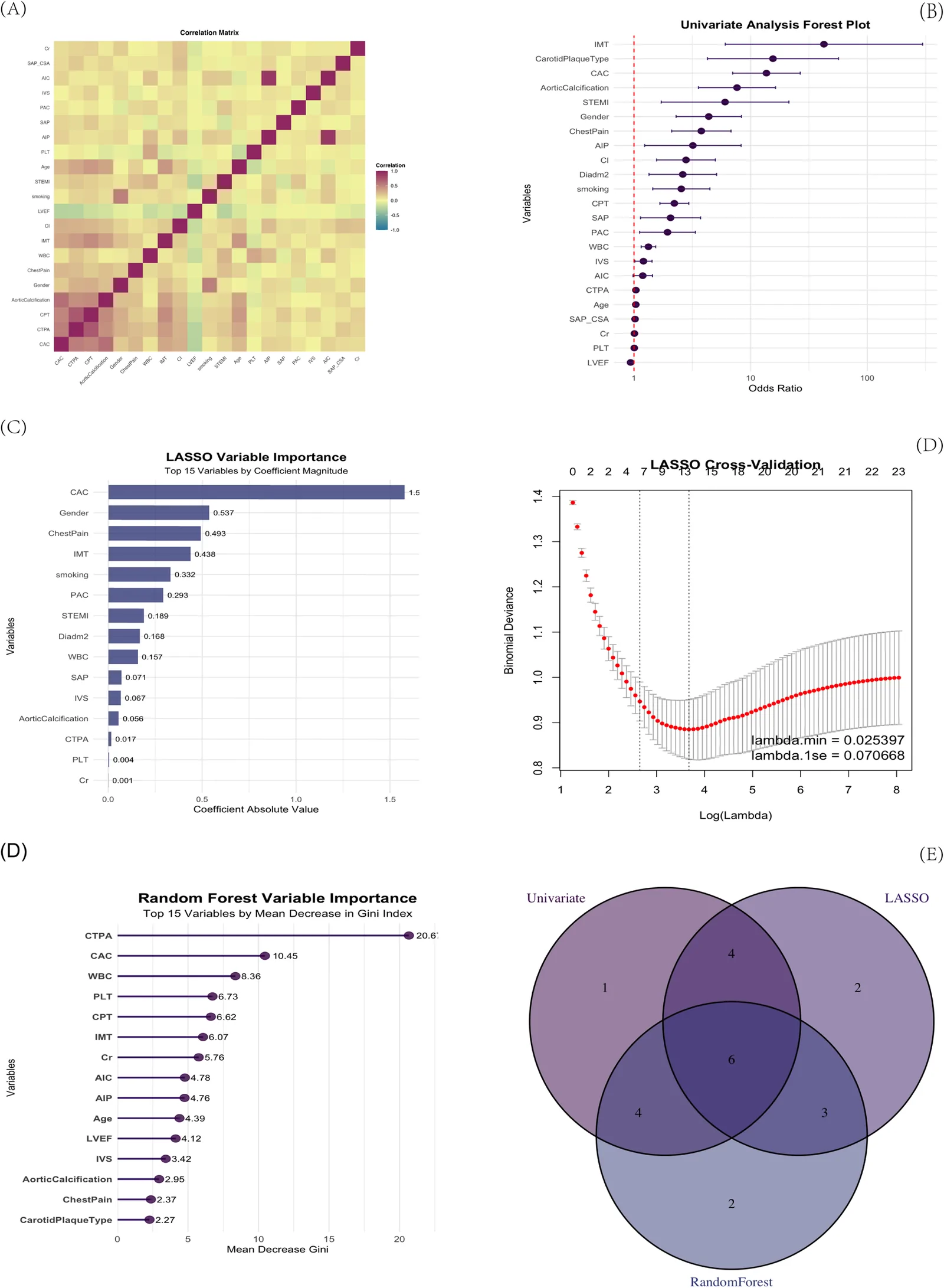
Feature selection plot. **(A)** Heatmap of variable importance; no Spearman correlation coefficients greater than 0.8 were observed. **(B)** Ranking chart of variables by importance from univariate logistic regression. **(C)** Forest plot of LASSO regression coefficients. **(D)** LASSO regularization path; variables between lambda.min and lambda.1se were most suitable for model building (range, 6–13 variables). **(E)** Variable importance selection chart from Random Forest. **(F)** Venn diagram; select six common variables from the feature sets of the three machine learning algorithms as the most important features.

The outcome of this study was a binary classification problem. Gensini score independently predicted stent implantation (OR = 1.07, 95% CI: 1.05–1.08, *P* < 0.001), confirming its reliability as a robust measure of coronary lesion severity. The Gensini coronary lesion quantification score was calculated from coronary angiography data and images under specialist guidance, and the calculated results were verified three times or more (16, 17). Calculated using the quartile algorithm (mean 31.61, median 27.00, P25 = 2.75, P75 = 52.00). Based on these distribution characteristics, we have adopted the median (27 points) as the cutoff value for binary classification in the revised manuscript, defining patients with a Gensini score <27 as the low-risk group and those with a score ≥27 as the high-risk group. A higher Gensini score indicates a more severe coronary artery disease (18, 19).

### 2.4 Baseline data and model development

The internal training set was randomly divided into a training set (70%) and a test set (30%) using random sampling, with a random seed (seed = 123) set to ensure experimental reproducibility. Model performance was primarily evaluated using the Receiver Operating Characteristic (ROC) curve, the area under the ROC curve (AUC), and its 95% confidence interval. Model performance was further assessed through ten-fold cross-validation and temporal validation, and interpretability was analyzed using SHapley Additive exPlanations (SHAP).

In the feature screening process, we initially used univariate logistic regression (LR), LASSO regression, and Random Forest (RF) for joint screening. LR enables preliminary feature screening by analyzing the relationship between each individual variable and the target variable to help identify the most significant features (20). The LASSO regression algorithm achieves variable selection through L1 regularization, effectively handling multicollinearity by shrinking unimportant regression coefficients to zero, making it particularly suitable for high-dimensional data and improving model interpretability through automatic feature selection (21). RF is a machine learning method capable of handling complex nonlinear relationships; its built-in out-of-bag (OOB) evaluation helps prevent overfitting and makes it robust to outliers (22, 23).

We developed models using eight machine learning algorithms: eXtreme Gradient Boosting (XGBoost), Logistic Regression (LR), Random Forest (RF), Support Vector Machine (SVM), K-Nearest Neighbors (KNN), Artificial Neural Network (ANN), Naïve Bayes (NB), and Gradient Boosting Machine (GBM). Calibration was assessed using calibration curves, the Brier score, the calibration intercept, the calibration slope, and the Spiegelhalter *z*-test. A well-calibrated model is defined as one with a Brier score <0.25 (Supplementary Material S2).

### 2.5 Statistical methods

Data entry and management were completed using Microsoft Excel 2021 (Microsoft Corp., Redmond, WA, USA). All statistical analyses and modeling were performed using R version 4.5.2 (R Foundation for Statistical Computing, Vienna, Austria), with the significance level set at two-sided *P* < 0.05.

## 3 Results

### 3.1 Characteristics of the study population

A total of 400 patients were identified in this study, Supplementary Material S1 presents the characteristics of the training set and validation sets, with no significant differences observed between the groups.

As shown in Figure 2, for the 28 clinical feature variables finally included in this study that are related to coronary artery lesions, three machine learning algorithms—univariate logistic regression (Figure 2B), LASSO regression (Figures 2C, 1D), and random forest (Figure 2E)—were used to select the top 15 most important feature variables in the training set (*n* = 210), respectively. Using a Venn diagram (Figure 2F), six common feature variables shared by all three algorithms were identified. To reach a total of ten, additional variables were sequentially added based on their calculated feature importance scores. Ultimately, the top ten variables included IMT, coronary and aortic calcification, CTPA, CPT, WBC, PLT, and smoking history, among others.

### 3.2 Model performance

As shown in Figure 3A, modeling was performed using eight machine learning models (Figure 3B), yielding the following AUCs: GBM (AUC = 0.891), XGBoost (AUC = 0.889), KNN (AUC = 0.812), Logistic Regression (AUC = 0.860), Naive Bayes (AUC = 0.870), Neural Network (AUC = 0.849), Random Forest (AUC = 0.884), and SVM (AUC = 0.850). Detailed model performance metrics are presented in Table 1. The performance of the GBM and XGBoost models was comparable.

**Figure 3:**
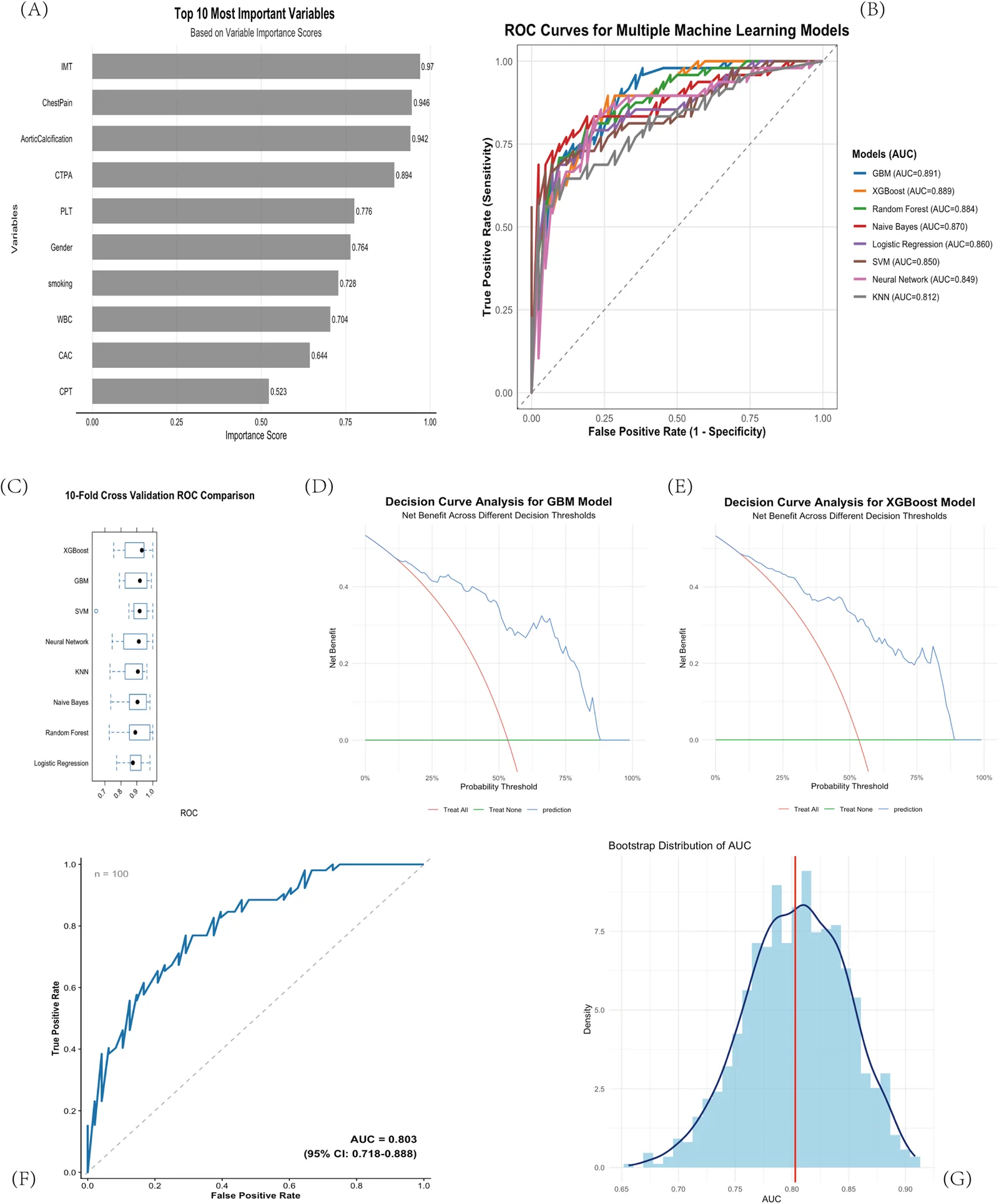
Diagram of predictive model development and validation. **(A)** Ranking of the top ten most important features in the predictive model. Features were selected using logistic regression, LASSO regression, and random forests, and then ranked by importance score to identify the top ten. **(B)** ROC curves for eight machine learning models. **(C)** Average area under the ROC curve (AUC) from 10-fold cross-validation. The closer the box is to the right, and the further the marker is to the right, the higher the model's stability and performance. **(D)** DCA curve for the GBM model (the DCA curve is used to validate and evaluate the performance of predictive models). As shown in the figure, when the threshold exceeds 60%, the net benefit declines rapidly and approaches the zero line (Treat None) near 75%, indicating that the model's decisions do not yield clinical net benefit at this point. **(E)** For the XGBoost model, within the relatively wide threshold range of 20%–80%, the blue curve (model net benefit) consistently remains above the two reference lines “Treat All” and “Treat None,” indicating that the model can deliver sustained clinical net benefit within this threshold range. **(F)** Independent temporal validation ROC curve for the XGBoost model. **(G)** Bootstrap sampling method (1,000 repetitions). The results show that the Bootstrap distribution of the AUC is approximately normal and exhibits a clear central tendency, indicating that the model has stable performance and strong generalization ability.

**Table 1:** Evaluation metrics for eight machine learning predictive models.

Model	AUC	Accuracy	Sensitivity	Specificity	Precision	F1
GBM	0.8906	0.7778	0.8125	0.7381	0.7800	0.7959
XGBoost	0.8889	0.8000	0.8333	0.7619	0.8000	0.8163
Random Forest	0.8839	0.7889	0.8125	0.7619	0.7959	0.8041
Naive Bayes	0.8695	0.7111	0.8333	0.5714	0.6897	0.7547
Logistic Regression	0.8596	0.7778	0.7292	0.8333	0.8333	0.7778
SVM	0.8497	0.7556	0.7292	0.7857	0.7955	0.7609
Neural Network	0.8487	0.8000	0.7917	0.8095	0.8261	0.8085
KNN	0.8118	0.7222	0.7708	0.6667	0.7255	0.7475

Comparison of the performance of eight machine learning models in predicting the risk of coronary heart disease using the test dataset. GBM, Gradient Boosting Machine; SVM, Support Vector Machine; KNN, K-Nearest Neighbors. The GBM model achieved the highest AUC (0.8906), while XGBoost and Neural Network tied for the highest accuracy (0.8000).

#### 3.2.1 Internal validation and clinical performance assessment

In 10-fold cross-validation, XGBoost demonstrated the highest stability (Figure 3C). The model showed satisfactory calibration (Brier score = 0.128), and the DeLong test confirmed its superior performance over most other machine learning models (*P* < 0.05, Supplementary Material S2–S3). Decision curve analysis (DCA) revealed a broader net benefit range than GBM, with the benefit gradually declining to zero only after the threshold probability exceeded 80% (Figures 3E–F). These findings support XGBoost as a reliable risk stratification tool with favorable discriminatory power, calibration, and clinical utility.

#### 3.2.2 Temporal validation

To verify the generalization ability of the model, this study included 100 patients from the same center between August and December 2025 as an independent temporal validation cohort, comprising 52 cases in the high-risk coronary heart disease group and 48 cases in the low-risk group. The temporal validation results showed that the XGBoost model demonstrated robust discriminatory power in this cohort, with an AUC value of 0.803 (95% CI: 0.718–0.888), as shown in Figure 3F. Bootstrap resampling showed that the AUC followed an approximately normal distribution with a clear central tendency; the adjusted AUC was 0.81 (95% CI: 0.73–0.88), indicating that the model performed consistently across resampling runs and demonstrated good generalization ability (Figure 3G). This provides reliable evidence to support subsequent clinical translation.

#### 3.2.3 Global model interpretation

Global interpretation based on SHAP values (see Figure 4A) revealed that CTPA served as the primary feature driving the prediction of moderate-to-severe coronary artery lesions. Figure 4B shows that these features contributed in a monotonically positive manner, while Figure 4C illustrates the correspondence between the actual values of key features and their corresponding SHAP values. The computational model is publicly available at: (https://zyy990726.shinyapps.io/XGB-model/).

**Figure 4:**
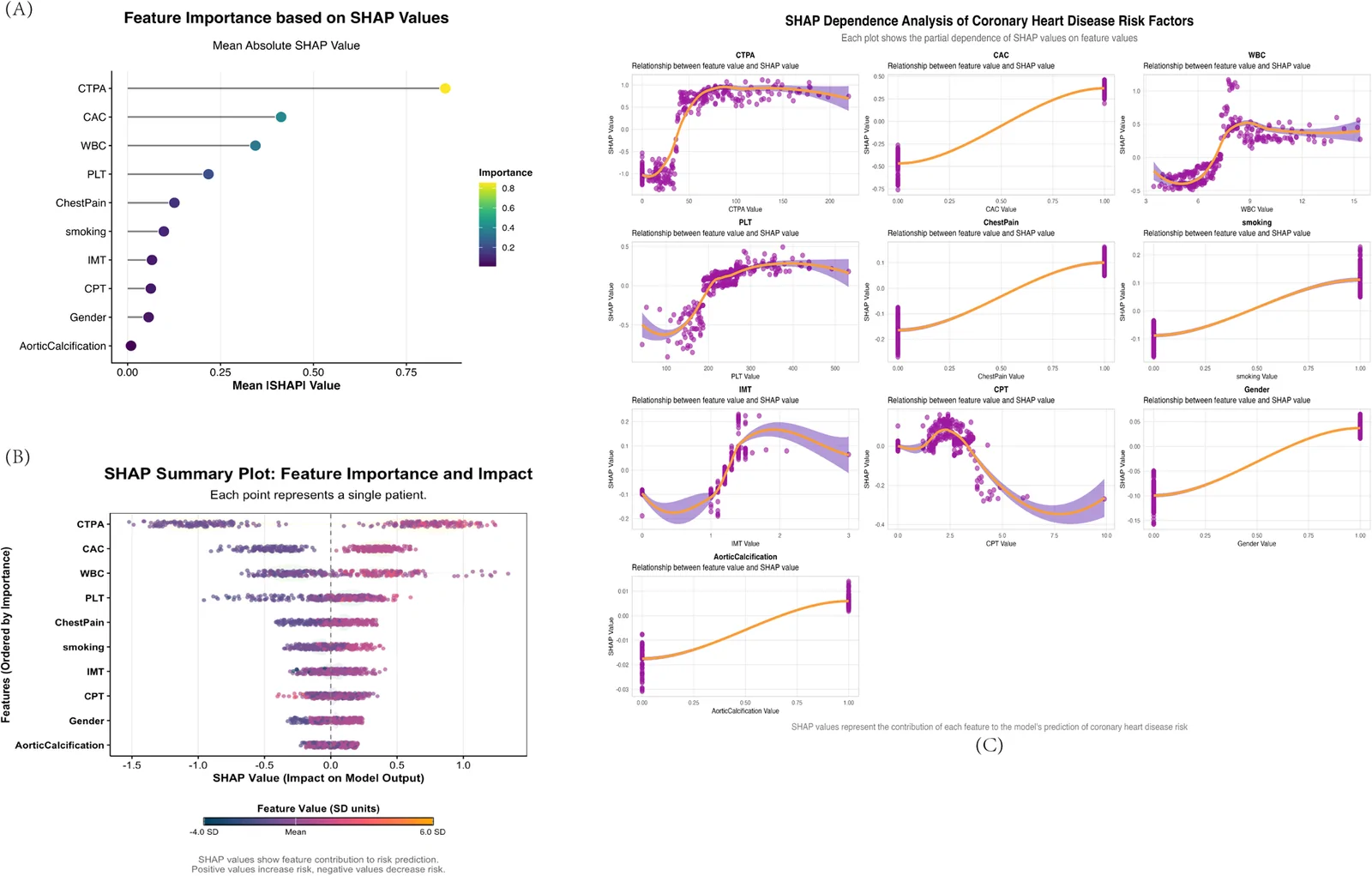
SHAP analysis chart. **(A)** SHAP Specialized Variable Importance Ranking Plot: This figure illustrates the contribution of each feature in the XGBoost model to predicting severe coronary artery lesions, ranked from highest to lowest. **(B)** SHAP Feature Heatmap: The vertical axis is sorted from highest to lowest by feature importance (mean absolute SHAP value), while the horizontal axis represents the SHAP value, indicating the direction and magnitude of each feature's influence on the model output (predicted probability). Each point represents a patient, and the color of the point indicates the level of the feature value (red for high values, blue for low values, after standardization). The figure shows that carotid plaque area (CTPA) is the most important predictor; high values are associated with higher SHAP values (indicating a positive prediction of moderate-to-severe lesions), with the remaining features following in order of importance. All features exhibit a clear dose-response relationship, meaning that the higher the feature value, the greater the likelihood of a positive contribution to the model output. **(C)** This figure illustrates the contribution of each predictor to the risk of moderate-to-severe coronary artery disease in the XGBoost model. CTPA, carotid plaque area; CAC, coronary artery calcification; WBC, white blood cell count; PLT, platelet count; IMT, carotid intima-media thickness; CPT, carotid plaque thickness; SHAP, Shapley Additive Explanations.

#### 3.2.4 Nonlinear association between carotid lesions and coronary artery disease severity

Restricted cubic spline analysis revealed a significant nonlinear relationship between CTPA and the Gensini score (overall *P*-value < 0.001; nonlinear *P*-value < 0.001; Figure 5A). The curve exhibits an inflection point at CTPA = 44; beyond this value, higher CTPA values correlate with greater severity of coronary artery disease (CAD). A second inflection point occurs at 123.2, marking the curve's entry into a plateau phase, which may indicate that atherosclerosis has reached a systemic, diffuse, and advanced stage. Similarly, CPT and the Gensini score also exhibit a significant nonlinear association (*P*_overall < 0.001; *P*_nonlinear = 0.004; Figure 5C), with an inflection point at 2.3 mm. Notably, the thresholds for both CTPA (Figure 5B) and CPT (Figure 5D) remained largely unchanged after adjusting for conventional cardiovascular risk factors, confirming their robustness.

**Figure 5:**
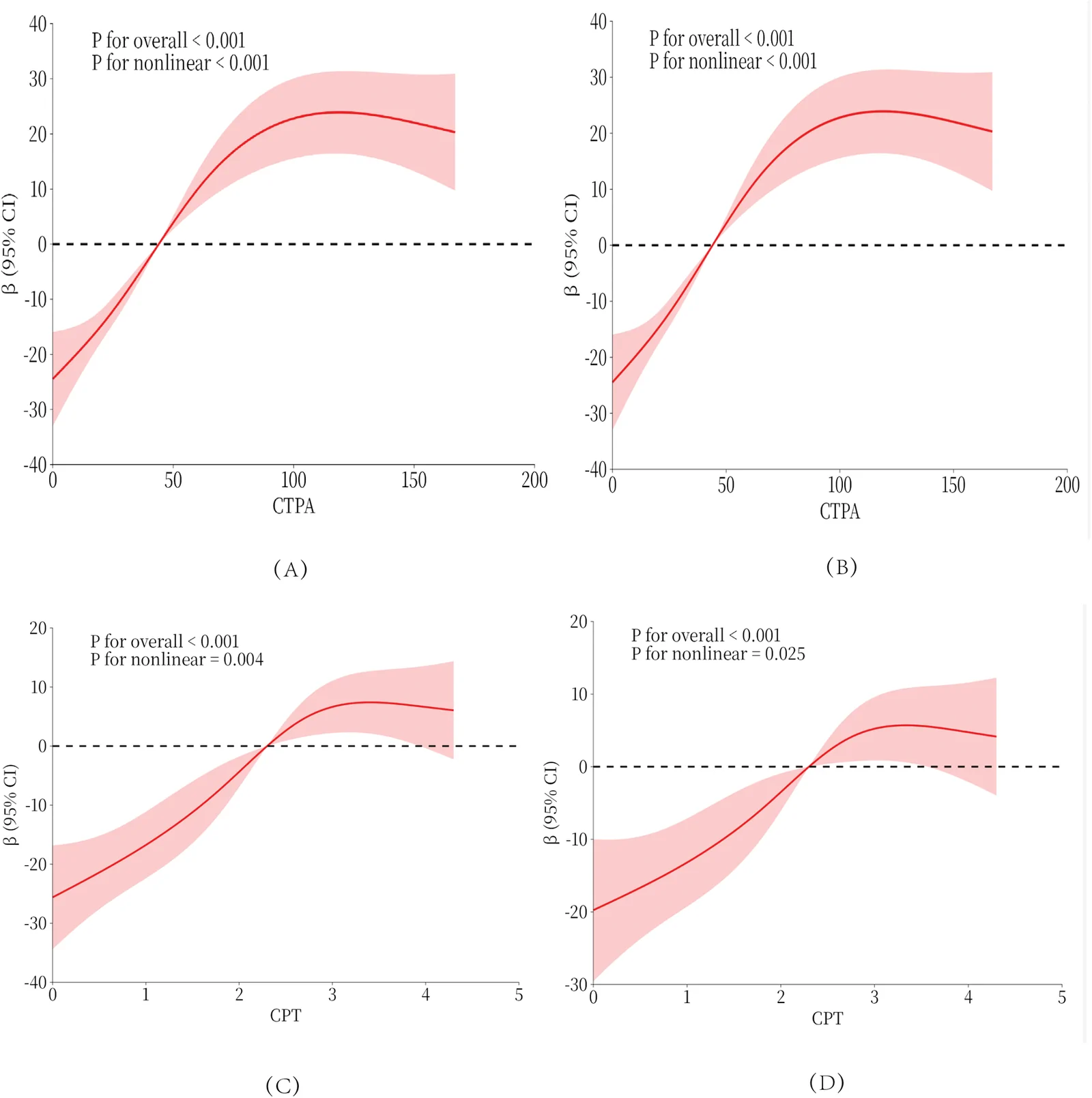
**(A)** Unadjusted restricted cubic spline (RCS) curves for carotid plaque area and Gensini scores. **(B)** Adjusted restricted cubic spline (RCS) curves for carotid plaque area and Gensini scores **(C)** Unadjusted restricted cubic spline (RCS) curves for carotid plaque thickness and Gensini scores. **(D)** Adjusted restricted cubic spline (RCS) curves for carotid plaque thickness and Gensini scores.

## 4 Discussion

In this retrospective cohort study, we systematically compared the performance of eight machine learning algorithms in predicting the risk of coronary artery lesions and constructed a predictive model based on XGBoost. The results showed that the XGBoost model achieved an AUC of 0.889 (95% CI: 0.819–0.945) in the internal validation and an AUC of 0.803 (95% CI: 0.718–0.888) in the independent temporal validation. Calibration assessment demonstrated good agreement between predicted probabilities and observed outcomes, with a Brier score of 0.128, an intercept of −0.167, a slope of 1.537, and a mean absolute error of 0.067. Although the Spiegelhalter test was significant (*P* < 0.001), the calibration curve and Emax (0.112) suggested that the magnitude of miscalibration was clinically acceptable (Supplementary Material S2).

The SHAP analysis identified 10 key predictors, including carotid plaque area, carotid plaque thickness, intima-media thickness, and coronary artery calcification. These independent risk factors were strongly associated with the severity of coronary artery disease. Variables and outcome events are correlated, but there is no causal relationship between them. The independent risk factors identified through Logistic regression in this study provide applicable risk quantification indicators for clinical use (Table 2). However, the 22-variable confirmatory logistic regression model (Table 2) was fitted using 157 high-risk events from the full internal set (*n* = 300), resulting in an event-per-variable (EPV) ratio of approximately 7.1, which is below the recommended threshold of 10 (24). Under these EPV conditions, regression coefficients and their confidence intervals may lack stability and be more prone to overfitting; however, the predictive variables that achieved statistical significance in this model (such as CTPA, CPT, and WBC) were consistently selected by three independent feature selection methods (univariate logistic regression, LASSO, and random forests), which supports the robustness of these associations. We will seek further validation in a larger multicenter cohort in the future.

**Table 2:** Correlation between the severity of coronary artery disease and characteristic variable (full internal set *n* = 300).

Variables	*β*	SE	*Z*	*P*		OR (95% CI)
Gender						
0						1.00 (Reference)
1	1.35	0.27	5.03	<0.001		3.84 (2.27∼6.49)
Smoking						
0						1.00 (Reference)
1	0.91	0.24	3.74	<.001		2.48 (1.54∼3.99)
Aortic calcification						
0						1.00 (Reference)
1		1.77	0.29	6.06	<.001	5.87 (3.31∼10.41)
CAC						
0						1.00 (Reference)
1		2.27	0.27	8.40	<.001	9.68 (5.70∼16.44)
Chest pain						
0						1.00 (Reference)
1		1.28	0.25	5.16	<.001	3.61 (2.22∼5.88)
Chest tightness						
0						1.00 (Reference)
1		0.46	0.32	1.44	0.149	1.58 (0.85∼2.92)
CTPA (mm^2^)		0.04	0.00	7.99	<.001	1.04 (1.03∼1.05)
CPT (mm)		0.71	0.12	6.09	<.001	2.03 (1.62∼2.55)
IMT (mm)		3.38	0.79	4.30	<.001	29.33 (6.28∼137.00)
TG (mg/dl)		0.27	0.12	2.27	0.023	1.31 (1.04∼1.66)
CHO (mg/dl)		0.16	0.09	1.76	0.079	1.17 (0.98∼1.40)
RBC		0.04	0.07	0.62	0.539	1.04 (0.92∼1.18)
WBC		0.29	0.06	4.86	<.001	1.34 (1.19∼1.51)
PLT		0.01	0.00	2.56	0.010	1.01 (1.01∼1.01)
HDLC (mg/dl)		−0.82	0.46	−1.77	0.076	0.44 (0.18∼1.09)
LDLC (mg/dl)		0.02	0.07	0.28	0.783	1.02 (0.88∼1.18)
AIP		1.31	0.42	3.09	0.002	3.70 (1.61∼8.50)
AIC		0.22	0.09	2.42	0.015	1.24 (1.04∼1.48)
Apob Apoa		0.20	0.51	0.39	0.693	1.22 (0.45∼3.36)
UA (mg/dl)		0.00	0.00	1.02	0.309	1.00 (1.00∼1.00)
Cr (mg/dl)		0.01	0.01	2.21	0.027	1.01 (1.01∼1.02)
Age (years)		0.03	0.01	2.79	0.005	1.03 (1.01∼1.05)

Multivariable logistic regression model fitted on the Full Internal Set (*n* = 300) with 22 predictor variables and 157 outcome events (high-risk cases), yielding an events-per-variable (EPV) ratio of approximately 7.1 (157/22). The table presents independent predictors of high-risk coronary artery disease, adjusting for potential confounding among variables. It also confirms that key features identified by XGBoost—including CTPA, CPT, and WBC—remain statistically significant in the regression model.

OR > 1: This variable is an independent risk factor for moderate-to-severe disease.

95% CI does not include 1: the difference is statistically significant (*P* < 0.05).

All variables included in this retrospective study are pathophysiologically correlated with coronary artery lesions. First, systemic vascular disease is a unified entity, and pan-vascular disease is a group of vascular system diseases sharing atherosclerosis as a common pathological feature (25, 26). The evaluation data of carotid artery lesions obtained through carotid color Doppler ultrasound—including carotid plaque area, carotid plaque thickness, and carotid intima-media thickness—represent the most straightforward, highly feasible, and cost-effective methods currently available in clinical practice. Carotid artery lesions hold significant value for predicting coronary artery lesions. Secondly, white blood cells also play an important role in the pathogenesis of inflammation, which is the initial key step in the formation of atherosclerotic plaques (27, 28). The Atherosclerosis Risk in Communities (ARIC) study has repeatedly confirmed that an elevated circulating white blood cell count is an independent predictor of future myocardial infarction, stroke, and cardiovascular death, with a predictive value surpassing traditional risk factors. This directly links leukocyte-mediated systemic inflammatory burden to clinical outcomes (29). Furthermore, platelets are major amplifiers of acute inflammation. In the early stages of vascular endothelial dysfunction, subendothelial matrix components such as collagen are exposed, and circulating platelets rapidly adhere via their surface glycoprotein receptors (e.g., GPVI, GPIb-IX-V) to form acute thrombi (27, 30). Gawaz M also detailed the correlation between platelet activity, the levels of platelet-monocyte aggregates, and the severity and prognosis of coronary heart disease (31). Studies have shown that, before menopause, the average age of onset of ASCVD in women is about 10 years later than in men, with a significantly lower incidence rate. This protective effect is primarily attributed to the vascular biological actions of endogenous estrogen (31). Research by Regitz-Zagrosek et al. has also confirmed the evidence of sex differences in epidemiology, mechanisms, and clinical prognosis (32). Smoking is the most potent and distinctly preventable pathogenic factor for ASCVD. Celermajer DS et al. were the first to use non-invasive ultrasound quantification to confirm that smoking (even in small amounts) can cause direct damage to vascular endothelial cells (33). Nicotine and carbon monoxide (CO) can inhibit endothelial nitric oxide synthase (eNOS), reducing the synthesis and bioavailability of nitric oxide (NO), which has vasodilatory, anti-inflammatory, and anti-proliferative effects. This is the initiating step and core defect in vascular lesions (30). Finally, we included atherosclerosis and calcification of the aorta and coronary arteries observed via chest CT images as predictors because they are not isolated local lesions but specific manifestations of systemic atherosclerosis in different vascular beds, which are independently associated with the risk of CHD and stroke (34). Detrano R and Budoff MJ et al. also confirmed a clear correlation between coronary artery calcification and CHD (35, 36). Therefore, the predictors ultimately included in this study underwent rigorous statistical screening and clinical significance evaluation, demonstrating stable contributions in both univariate analysis and multivariate regularized feature selection, which confirms the validity and reliability of the selected features. The predictive model built upon these features exhibited good discrimination, calibration, and generalization abilities, further verifying the model's robustness and clinical applicability.

This study has certain limitations. First, the study was designed as a single-center retrospective cohort. Although data from a different time period were used as the temporal validation set, all patients were from the same medical center, meaning that we cannot rule out the institutional and detection bias associated with this single-center temporal validation design. The generalization ability of the model still needs to be further validated in prospective cohorts across multiple centers and diverse populations in the future. Second, the sample size was relatively limited (*n* = 400), with the temporal validation set containing only 100 cases, which might lead to wider confidence intervals for performance estimates. Third, this study utilized the Gensini score as the sole endpoint and did not conduct long-term follow-up for hard clinical events such as myocardial infarction, cardiac death, or revascularization. The current model can only predict the severity of angiographic lesions and cannot predict prognostic outcomes, which limits its direct clinical utility. We have initiated a prospective follow-up cohort study to collect long-term major adverse cardiovascular event (MACE) data, and we will evaluate the prognostic performance of this model in subsequent research. However, we plotted Restricted Cubic Spline (RCS) curves for carotid-related predictor variables against the Gensini score and performed correlation analysis, verifying the correlation between carotid lesions and coronary lesions. Future studies could further expand the data sample size to construct a continuous prediction model, thereby improving prediction accuracy. Fourth, subgroup analysis for model fairness was not conducted. Future studies need to evaluate the consistency of model performance across different sex and age subgroups.

In summary, this model demonstrates a high degree of reliability in terms of both its theoretical foundation and practical validation, providing valuable guidance for clinical decision-making. This tool is expected to assist clinicians in developing risk stratification and intervention strategies for coronary artery disease, driving a shift in predictive modeling from a focus solely on “high performance” to achieving “high clinical value,” and providing decision support for the early identification and management of coronary artery disease.

## 5 Conclusion

This study established and validated a coronary artery lesion risk prediction model based on the XGBoost algorithm. Internal and temporal validation results confirmed its excellent discriminatory power and clinical utility. Future studies could further validate the model's generalization ability through prospective, multicenter clinical trials and quantitatively evaluate its accuracy in improving clinical decision-making and patient outcomes.

## Data Availability

The raw data supporting the conclusions of this article will be made available by the authors, without undue reservation.
