# Supplementary material for: Development and validation of a machine learning model for predicting coronary artery disease risk: a retrospective study
Source: Front Cardiovasc Med. 2026 Sep 22;13:1874227. doi: 10.3389/fcvm.2026.1874227 (PMC13640014; doi:10.3389/fcvm.2026.1874227)
Supplement: Supplementary file 1 [file Supplementaryfile1.docx]

Supplementary Material

# 1 Supplementary Tables

Supplement 1 Comparison of demographic and clinical characteristics between training set and internal validation set

| Variables | test (n = 90) | train (n = 210) | Statistic | P |  |
| --- | --- | --- | --- | --- | --- |
|  |  |  |  |  |  |
| CPA，mm^2^ | 52.50 (15.05, 87.75) | 40.00 (14.45, 76.75) | Z=-1.35 | 0.178 |  |
| CarotidPlaqueThickness,  mm, | 2.55 (1.60, 3.08) | 2.30 (1.52, 2.77) | Z=-1.66 | 0.096 |  |
| IMT, mm, | 1.30 (1.20, 1.40) | 1.20 (1.20, 1.40) | Z=-1.45 | 0.147 |  |
| CarotidArteryStenosis,% | 0.00 (0.00, 0.00) | 0.00 (0.00, 0.00) | Z=-0.86 | 0.388 |  |
| SA CSA, mm^2^ | 0.00 (0.00, 0.00) | 0.00 (0.00, 0.00) | Z=-0.96 | 0.337 |  |
| IVST, mm | 10.00 (9.00, 11.00) | 10.00 (9.00, 11.00) | Z=-0.47 | 0.636 |  |
| E cm/s | 0.70 (0.59, 0.80) | 0.69 (0.56, 0.84) | Z=-0.29 | 0.773 |  |
| A, cm/s | 0.86 (0.74, 0.98) | 0.82 (0.67, 0.95) | Z=-1.52 | 0.128 |  |
| E A | 0.75 (0.65, 1.14) | 0.79 (0.68, 1.22) | Z=-0.94 | 0.347 |  |
| LVEDD | 45.00 (39.25, 50.00) | 44.00 (39.25, 48.75) | Z=-0.84 | 0.402 |  |
| LVEF, % | 65.50 (61.00, 69.00) | 66.00 (61.00, 69.75) | Z=-0.05 | 0.957 |  |
| WBC，*109 | 6.78 (5.67, 8.86) | 6.99 (5.68, 8.69) | Z=-0.44 | 0.662 |  |
| RBC，*109 | 4.54 (4.24, 4.87) | 4.54 (4.20, 4.98) | Z=-0.43 | 0.670 |  |
| PLT,*109 | 219.50 (181.25, 262.75) | 241.00 (193.25, 277.75) | Z=-2.41 | 0.016 |  |
| CHO, mmol/L | 4.67 (3.99, 5.55) | 4.84 (4.06, 5.70) | Z=-0.41 | 0.684 |  |
| TG, mmol/L | 1.44 (1.02, 2.02) | 1.52 (1.11, 1.94) | Z=-0.53 | 0.599 |  |
| HDLC, mmol/L | 1.10 (0.80, 1.30) | 1.00 (0.88, 1.18) | Z=-1.29 | 0.198 |  |
| LDLC, mmol/L | 2.59 (1.80, 3.30) | 2.63 (1.90, 3.30) | Z=-0.66 | 0.507 |  |
| Apoa,G/L | 1.10 (1.00, 1.30) | 1.17 (1.00, 1.30) | Z=-0.96 | 0.339 |  |
| Apob,G/L | 0.83 (0.70, 1.00) | 0.90 (0.70, 1.00) | Z=-1.37 | 0.172 |  |
| AIP | 0.12 (-0.07, 0.38) | 0.17 (0.02, 0.35) | Z=-1.19 | 0.235 |  |
| AIC | 0.31 (-0.16, 1.39) | 0.47 (0.04, 1.22) | Z=-1.19 | 0.235 |  |
| Apob Apoa | 0.74 (0.60, 0.87) | 0.77 (0.65, 0.91) | Z=-1.00 | 0.320 |  |
| Lp a, mg/dL | 151.14 (74.00, 233.00) | 134.70 (85.57, 234.00) | Z=-0.12 | 0.903 |  |
| UA, umol/L | 352.00 (284.00, 405.00) | 365.00 (304.25, 431.00) | Z=-1.14 | 0.255 |  |
| Cr, umol/L | 77.50 (66.70, 90.45) | 76.55 (64.00, 88.00) | Z=-0.53 | 0.597 |  |
| PT, s | 11.25 (10.83, 11.90) | 11.40 (10.90, 12.10) | Z=-1.26 | 0.209 |  |
| FDP, g/L | 2.40 (1.90, 2.80) | 2.15 (1.80, 2.80) | Z=-1.28 | 0.200 |  |
| D-dimer, g/L | 0.39 (0.24, 0.66) | 0.34 (0.20, 0.58) | Z=-0.93 | 0.353 |  |
| Smoking, n(%) |  |  | χ²=0.04 | 0.838 |  |
| 0 | 52 (57.78) | 124 (59.05) |  |  |  |
| 1 | 38 (42.22) | 86 (40.95) |  |  |  |
| STEMI, n(%) |  |  | χ²=0.59 | 0.444 |  |
| 0 | 82 (91.11) | 185 (88.10) |  |  |  |
| 1 | 8 (8.89) | 25 (11.90) |  |  |  |
| NSTREMI, n(%) |  |  | χ²=0.05 | 0.828 |  |
| 0 | 78 (86.67) | 180 (85.71) |  |  |  |
| 1 | 12 (13.33) | 30 (14.29) |  |  |  |
| SAP, n(%) |  |  | χ²=2.10 | 0.147 |  |
| 0 | 59 (65.56) | 155 (73.81) |  |  |  |
| 1 | 31 (34.44) | 55 (26.19) |  |  |  |
| UAP, n(%) |  |  | χ²=1.05 | 0.306 |  |
| 0 | 73 (81.11) | 159 (75.71) |  |  |  |
| 1 | 17 (18.89) | 51 (24.29) |  |  |  |
| Gender， n(%) |  |  | χ²=0.36 | 0.550 |  |
| Male | 64 (71.11) | 142 (67.62) |  |  |  |
| Female | 26 (28.89) | 68 (32.38) |  |  |  |
| Hypertension， n(%) |  |  | χ²=2.87 | 0.090 |  |
| 0 | 20 (22.22) | 67 (31.90) |  |  |  |
| 1 | 70 (77.78) | 143 (68.10) |  |  |  |
| Diadm2， n(%) |  |  | χ²=3.28 | 0.070 |  |
| 0 | 74 (82.22) | 152 (72.38) |  |  |  |
| 1 | 16 (17.78) | 58 (27.62) |  |  |  |
| Heart Failure ， n(%) |  |  | χ²=2.15 | 0.142 |  |
| 0 | 60 (66.67) | 121 (57.62) |  |  |  |
| 1 | 30 (33.33) | 89 (42.38) |  |  |  |
| Valvular Heart Disease， n(%) |  |  | χ²=0.34 | 0.558 |  |
| 0 | 87 (96.67) | 197 (94.26) |  |  |  |
| 1 | 3 (3.33) | 12 (5.74) |  |  |  |
| Liver Disease， n(%) |  |  | χ²=0.38 | 0.537 |  |
| 0 | 86 (95.56) | 195 (93.75) |  |  |  |
| 1 | 4 (4.44) | 13 (6.25) |  |  |  |
| Lung Disease， n(%) |  |  | χ²=0.07 | 0.785 |  |
| 0 | 69 (76.67) | 164 (78.10) |  |  |  |
| 1 | 21 (23.33) | 46 (21.90) |  |  |  |
| Malignant Tumor， n(%) |  |  | χ²=0.00 | 1.000 |  |
| 0 | 87 (96.67) | 201 (96.63) |  |  |  |
| 1 | 3 (3.33) | 7 (3.37) |  |  |  |
| Cerebral Infarction， n(%) |  |  | χ²=0.01 | 0.909 |  |
| 0 | 67 (74.44) | 155 (73.81) |  |  |  |
| 1 | 23 (25.56) | 55 (26.19) |  |  |  |
| Hyperlipidemia， n(%) |  |  | χ²=0.00 | 0.980 |  |
| 0 | 46 (51.11) | 107 (50.95) |  |  |  |
| 1 | 44 (48.89) | 103 (49.05) |  |  |  |
| Atrial Fibrillation， n(%) |  |  | χ²=1.07 | 0.302 |  |
| 0 | 85 (94.44) | 190 (90.91) |  |  |  |
| 1 | 5 (5.56) | 19 (9.09) |  |  |  |
| VPB， n(%) |  |  | χ²=0.03 | 0.859 |  |
| 0 | 51 (57.30) | 118 (56.19) |  |  |  |
| 1 | 38 (42.70) | 92 (43.81) |  |  |  |
| PAC，n(%) |  |  | χ²=0.02 | 0.888 |  |
| 0 | 47 (52.22) | 111 (53.11) |  |  |  |
| 1 | 43 (47.78) | 98 (46.89) |  |  |  |
| VPB， n(%) |  |  | - | 0.531 |  |
| 0 | 83 (92.22) | 199 (94.76) |  |  |  |
| 1 | 6 (6.67) | 10 (4.76) |  |  |  |
| 2 | 1 (1.11) | 1 (0.48) |  |  |  |
| CarotidPlaqueType, n(%) |  |  | χ²=2.73 | 0.435 |  |
| 0 | 15 (16.67) | 38 (18.10) |  |  |  |
| 1 | 7 (7.78) | 28 (13.33) |  |  |  |
| 2 | 63 (70.00) | 137 (65.24) |  |  |  |
| 3 | 5 (5.56) | 7 (3.33) |  |  |  |
| SubclavianArteryAtherosclerosis, n(%) |  |  | - | 0.717 |  |
| 0 | 77 (85.56) | 169 (80.48) |  |  |  |
| 1 | 13 (14.44) | 38 (18.10) |  |  |  |
| 50 | 0 (0.00) | 2 (0.95) |  |  |  |
| 69 | 0 (0.00) | 1 (0.48) |  |  |  |
| AorticCalcification, n(%) |  |  | χ²=0.61 | 0.435 |  |
| 0 | 23 (25.56) | 63 (30.00) |  |  |  |
| 1 | 67 (74.44) | 147 (70.00) |  |  |  |
| CAS, n(%) |  |  | χ²=2.96 | 0.085 |  |
| 0 | 34 (37.78) | 102 (48.57) |  |  |  |
| 1 | 56 (62.22) | 108 (51.43) |  |  |  |
| InternalCarotidArteryDisease, n(%) |  |  | χ²=1.33 | 0.249 |  |
| 0 | 7 (7.87) | 26 (12.44) |  |  |  |
| 1 | 82 (92.13) | 183 (87.56) |  |  |  |
| ChestPain, n(%) |  |  | χ²=0.09 | 0.761 |  |
| 0 | 36 (40.45) | 81 (38.57) |  |  |  |
| 1 | 53 (59.55) | 129 (61.43) |  |  |  |
| SOB, n(%) |  |  | χ²=0.29 | 0.588 |  |
| 0 | 76 (85.39) | 174 (82.86) |  |  |  |
| 1 | 13 (14.61) | 36 (17.14) |  |  |  |
| Chest Tightness, n(%) |  |  | χ²=0.35 | 0.555 |  |
| 0 | 16 (17.98) | 32 (15.24) |  |  |  |
| 1 | 73 (82.02) | 178 (84.76) |  |  |  |

Supplement1: This table presents a comparison of baseline data between the internal training set and the external validation set, and no significant differences were observed. Based on the data review, there were no significant intergroup differences in carotid artery lesions, cardiac echocardiography parameters, chest CT results, white blood cell count, red blood cell count, uric acid levels, or creatinine/prothrombin time (P > 0.05). Regarding clinical outcomes, no significant differences were observed between groups for hypertension, diabetes, arrhythmias, or malignant tumors.
