# Supplementary material for: Development and validation of a machine learning model for predicting coronary artery disease risk: a retrospective study
Source: Front Cardiovasc Med. 2026 Sep 22;13:1874227. doi: 10.3389/fcvm.2026.1874227 (PMC13640014; doi:10.3389/fcvm.2026.1874227)
Supplement: Supplementary file 2 [file Supplementaryfile2.docx]

Supplementary Material

# 1 Supplementary Tables2

Table 1 DeLong test results — pairwise comparison of AUC between XGBoost and other models

| **Model** | **AUC** | **AUC difference (vs XGBoost)** | **Z-value** | **P-value** | **Significance** |
| --- | --- | --- | --- | --- | --- |
| Random Forest | 0.947 | 0.0412 | 5.364 | <0.001 | Significant |
| SVM | 0.922 | 0.0161 | 1.611 | 0.1073 | Not significant |
| KNN | 0.917 | 0.0113 | 0.905 | 0.3657 | Not significant |
| XGBoost | 0.906 | — | — | — | — |
| Neural Network | 0.904 | –0.0022 | –0.186 | 0.8524 | Not significant |
| GBM | 0.881 | –0.0254 | –2.964 | 0.003 | Significant |
| Naive Bayes | 0.877 | –0.0288 | –2.451 | 0.0143 | Significant |
| Logistic Regression | 0.876 | –0.0301 | –2.395 | 0.0166 | Significant |

Note: AUC differences are calculated with XGBoost as the reference. Positive values indicate higher AUC than XGBoost; negative values indicate lower AUC than XGBoost. Significance level was set at P < 0.05.
