# Supplementary material for: Development and validation of a machine learning model for predicting coronary artery disease risk: a retrospective study
Source: Front Cardiovasc Med. 2026 Sep 22;13:1874227. doi: 10.3389/fcvm.2026.1874227 (PMC13640014; doi:10.3389/fcvm.2026.1874227)
Supplement: Supplementary file 3 [file Supplementaryfile3.docx]

Supplementary Material

# 1 Supplementary Figures

Abbreviations: C (ROC), area under the receiver operating characteristic curve; Dxy, Somers' D rank correlation; R², Nagelkerke's pseudo R-squared; D, model discrimination statistic; U, unexplained discrimination; Q, explained discrimination (D − U); Emax, maximum absolute calibration error; E90, 90th percentile of absolute calibration error; Eavg, mean absolute calibration error; S:z, Spiegelhalter z-test statistic; S:p, Spiegelhalter test P-value. Brier score < 0.25 indicates acceptable calibration accuracy; intercept and slope close to 0 and 1, respectively, indicate good calibration.
